# Statistical evaluation of tongue capability with visual feedback

**DOI:** 10.1186/s12984-023-01293-7

**Published:** 2024-01-02

**Authors:** Veronica Bratland, Kyle Coda, Mohamad Merei, Leila Ahmadian, Edna M. Babbitt, James. L. Patton, Hananeh Esmailbeigi

**Affiliations:** 1 Department of Biomedical Engineering, University of Illinois at Chicago, 218 SEO, 851 South Morgan Street, Chicago, IL 60607 USA; 2 Department of Electrical & Computer Engineering, University of Illinois at Chicago, 851 South Morgan Street, Chicago, IL 1020 SEO, 60607 USA; 3 Department of Restorative Dentistry, College of Dentistry, University of Illinois at Chicago, 801 S Paulina St, Chicago, IL 60612 USA; 4 Center for Aphasia Research and Treatment, Shirley Ryan AbilityLab, 355 E Erie St, Chicago, IL 60611 USA; 5 RobotLab, Center for Neuroplasticity, Shirley Ryan AbilityLab, 355 E Erie St, Chicago, IL 60611 USA; 6 Department of Computer Science, University of Illinois at Chicago, 11th floor SEO, 851 South Morgan Street, Chicago, IL 60607 USA

**Keywords:** Movement Analysis, Tongue Movement, Motor Control, Probability distribution analysis, Visual feedback, Diagnostic and Rehabilitation techniques, Wearable Technology, Intraoral devices

## Abstract

**Background:**

Analysis of tongue movement would benefit from a reference showcasing healthy tongue capability. We aimed to develop a reference of tongue capability and evaluated the role of visual feedback on the expression of movement.

**Methods:**

Using a wireless tracking intraoral wearable device, we composed probability distributions of the tongue tip as subjects were asked to explore the entire sensing surface area. Half of the 32 subjects received live visual feedback of the location of the center of the tongue tip contact.

**Results:**

We observed that the visual feedback group was 51.0% more consistent with each other in the position domain, explored 21.5% more sensing surface area, and was 50.7% more uniformly distributed. We found less consistent results when we evaluated velocity and acceleration.

**Conclusion:**

Visual feedback best established a healthy capability reference which can be used for designing new interfaces, quantifying tongue ability, developing new diagnostic and rehabilitation techniques, and studying underlying mechanisms of tongue motor control.

**Supplementary Information:**

The online version contains supplementary material available at 10.1186/s12984-023-01293-7.

## Introduction

The complex motor skills of the tongue are just one component of the array of fine motor movements required for vital tasks such as speech, breathing, mastication, and swallowing [1]. Assessing tongue movement capability may provide insights for quantifying impaired tongue ability, developing new diagnostic and rehabilitation techniques, designing new interfaces, and studying the underlying mechanisms of tongue control. In this study, we developed a methodology (comprising of task, device, feedback) for quantifying tongue movement, which we utilized to construct a description of tongue movement capabilities, which we refer to as a “*capability reference”*.

Studies of limb motor control have employed the free-exploration task to investigate movement distributions and construct descriptions of capability [2–6]. Free-exploration has been shown to encourage individuals to express their range of movement during self-directed explorations, providing a detailed reflection of an individual’s ability. While free-exploration has been applied to study various limb movements, tongue movement remains underexplored. However, free-exploration evaluations hold the potential to reveal abilities not discernible through clinical assessments, such as evaluations of tongue strength or rate of speech productions [7–9]. Here, we employed the free-exploration task to generate probability distributions of tongue movement, which formed the foundation for development of a capability reference.

To date, the most common devices for assessing the tongue are primarily focused on function over underlying capability, partially due to limitations in size and mobility of available technologies. Currently, tongue strength is measured using hand-held devices such as the Iowa-Oral-Pressure-Instrument (IOPI) and the Tongueometer [10–12]. Electromyography has been used to study tongue muscles activity mainly in neuromuscular diseases [13, 14]. Tongue movement has been assessed using imaging techniques, including magnetic resonance imaging, X-ray, ultrasound, and video-fluoroscopy, which provide visual representations of the tongue shape and position [15–19]. In addition, specialized tools designed for studying tongue movement include electromagnetic articulography (EMA) and electropalatography (EPG). EMA involves attaching multiple wired electromagnetic sensors to the articulators to create avatar-based models of the tongue’s movement [20], while EPG uses an artificial palate wired to an external recording system that visualizes real-time tongue-palate contacts [21]. Studies have shown that visual feedback of tongue shape and movement has led to functional improvement in swallowing and sound production [22–29]. However, interpreting visual representations produced by imaging technologies can be challenging for untrained individuals, and the visual plane of feedback has been shown to significantly impact motor adaptation [30–32].

To address the above-mentioned limitations (size, mobility, feedback), our group has developed the Tongue-Trackpad - a portable, wireless, tracking intraoral wearable device. The Tongue-Trackpad records the center of the tongue tip contact and provides real-time visual feedback of its movement [33–35]. In this study, we used the Tongue-Trackpad to compose tongue movement probability distributions during the free-exploration task, while we investigated the role of feedback on the expression of movement and consequently the capability reference. It is crucial to emphasize that free exploration of tongue tip contact is distinct from functional movements, as it exclusively isolates the tongue from all other motor movements necessary for executing functional tasks. However, assessments involving free exploration have demonstrated the potential to uncover capabilities that may not be apparent in evaluations of function [2–6].

Here, we first examined the amount of data required to reliably describe tongue probability distributions. Next, we investigated whether feedback influenced the movement expression (coverage area, bivariate kurtosis, and uniformity of the distributions). We then developed and tested the proposed capability reference. Our hypothesis was that the group receiving visual feedback would display higher inter-subject similarity, a broader coverage area, and greater uniformity in distribution, thus enabling the development of the capability reference.

## Methods

### Subjects

This study included 32 unimpaired subjects, 13 females and 19 males with an average age of 24.6 ± 4.9 years. Subjects provided informed consent in accordance with the University of Illinois at Chicago’s Institutional Review Board Protocol Number 2017 − 0550.

### Data acquisition

**Fig. 1:**
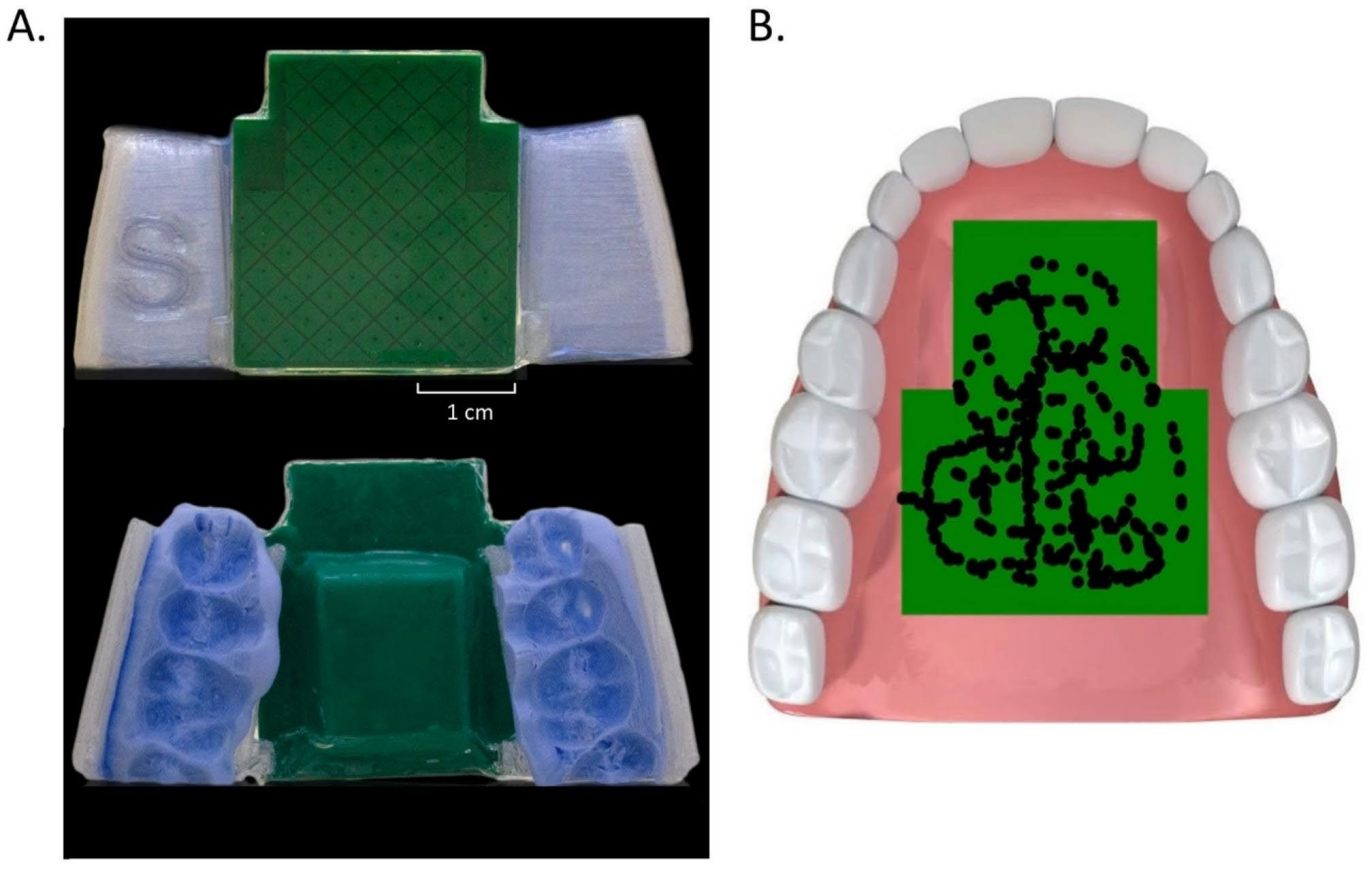
The Tongue-Trackpad and Visual Feedback Graphical User Interface. **(A)** The top image shows a size small (S) device with the diamond-shaped pads etched on the electronic board forming the active sensing area (shown in green). The device is hermetically sealed with thermoplastic. The bottom image shows the electronic board and a subject-specific impression of the upper teeth, acquired via the Bite Registration material. The personalized impression ensures a secure fit of the device to the teeth. **(B)** Customized graphical user interface displaying the exploration area of the device, the position of the center of the tongue tip contact is depicted by a 1-millimeter diameter dot. The cumulative history of the contacts is illustrated by previous dots, providing an overview of the path taken during exploration.

The Tongue-Trackpad, developed by our team was used to study tongue movement. The Tongue-Trackpad (Fig. 1) is a wireless intraoral device that detects tongue contact via capacitive sensing. The device’s sensing electronics board is incorporated inside a universal-fit retainer. The universal-fit retainer is designed in four sizes (X-Small, Small, Medium, Large) under the consultation of the University of Illinois at Chicago College of Dentistry to ensure safety and comfort. The device is hermetically sealed using biocompatible thermoplastic (Splint Material, Keystone Industries). Bite Registration Material (Vinyl Polysiloxane, Exabite II NDS) is utilized to capture precise dental impressions to securely fixate the device at the tooth-line.

The electronic module is powered via a rechargeable 3.7 V lithium-ion battery. The module’s printed circuit board (PCB) has two distinct sides with one side featuring a capacitive sensor matrix (trackpad) for detecting tongue movements, while the other side contains a Bluetooth Low Energy Central Processing Unit (BLE CPU) and essential electronic components. A ground hatch plane isolates the trackpad from electromagnetic interferences of the electronics. The trackpad surface is covered with diamond-shaped sensor pads, measuring 3 × 3 mm with a pitch of 4.5 mm, arranged in 8 rows and 7 columns. The diamond shape of the pads allows for a smooth transition of contact recording during a tongue swipe. The trackpad has an area of 720 mm^2^ and is narrower towards the front of the mouth to maximize coverage of the hard palate. Tongue contact is detected via self-capacitance, hence when the tongue contacts the trackpad, a grounded conductive plane is formed parallel to the contact area, resulting in a capacitance rise compared to the baseline, which is read by the CPU. The change in capacitance signals of the rows and columns are communicated via BLE to a custom graphical user interface.

Tongue contact with the trackpad is elliptical in shape, resulting in a Gaussian capacitive response centered at the midpoint of the contact. We used this characteristic to develop a saliva-tolerant algorithm, that would filter out any events that depict deviation from a Gaussian response which are attributed to saliva contact. The capacitive signals of the rows and columns are used to calculate the geometric center of the tongue contact. The trackpad has a spatial resolution of 1 mm, resulting in 720 resolvable contact positions. The trackpad’s mean positional accuracies, defined as the absolute value of the difference between real position and the device measured position, are 0.52 ± 0.48 mm (mean ± standard deviation) along the rows and 0.73 ± 0.62 mm along the columns.

### Experimental protocol

The study consisted of a single session which started with device fitting and dental impression acquisition. Subjects were seated at a comfortable distance of 2–3 feet from a computer screen and were instructed to perform the free-exploration task. In this task, participants were asked to continuously explore the Tongue-Trackpad sensing area with the tip of the tongue at a self-directed speed without lifting, while covering as much surface area as possible. The free-exploration task was performed in twenty 30-second exploration blocks (10 min total), each separated by a 30-second rest period to prevent fatigue, during which the device was removed, and the contact surface was cleared of saliva.

The visual feedback was presented to subjects using a customized graphical user interface developed in HyperText Markup Language (HTML) (Fig. 1B). The user interface displayed the exploration area along with a 1-millimeter diameter dot indicating the real-time position of the center of tongue tip contact, along with its cumulative exploration history. The cumulative position history was preserved across exploration blocks. The explored surface area was calculated at the conclusion of each block. To encourage a full profile of capability expression, the cumulative history was cleared when 80% of the available pixels in the graphical display was covered. Half of the subjects (6 females, 10 males, average age: 25.7 ± 5.6 years), received live visual feedback via the interface during the free-exploration task, we refer to this group as the visual feedback (VF) group. The remaining half of the subjects (7 females, 9 males, average age: 23.6 ± 4.2 years) were not presented with the visual feedback, we refer to this group as the without visual feedback (W/O VF) group.

### Data analysis

**Fig. 2:**
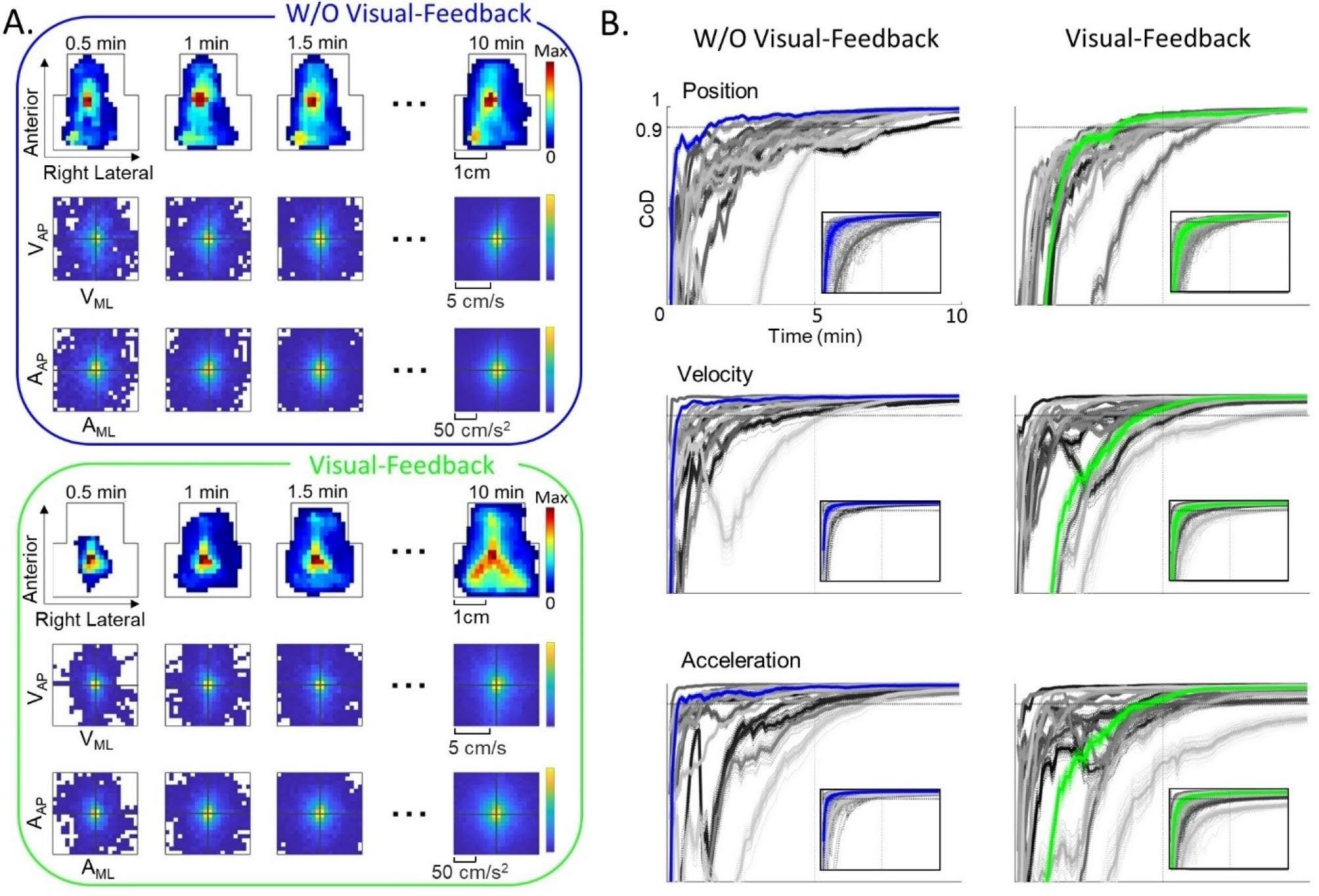
Temporal Evolution of Movement Probability Distributions During 10 min of Free-Exploration. **(A)** Cumulative probability distributions over time intervals (0-0.5 min, 0–1 min, etc.) illustrating position (top), velocity (middle), and acceleration (bottom) along the anteroposterior (AP) and mediolateral (ML) axes. Two representative subjects from the W/O VF and VF group are depicted by blue and green boarders, respectively. **(B)** Time to characterization, point at which the CoD reached 0.9, in the W/O VF group (left) and the VF group (right) for all subjects using successive epochs are presented. Blue and green curves correspond to the representative subjects in **(A)**, while the gray-scale curves represent all other subjects. Dashed lines indicate each of the 50 randomizations steps for each subject, whereas the solid lines denote the average of the 50 curves. The horizontal dashed line represents the 0.9 threshold. In the acceleration domain, one subject in the VF group did not reach the threshold for determination. Inserts in each plot display curves for random-order epochs. These results suggest that 5 min of data are sufficient to describe movement distributions across all kinematic domains

MATLAB (The MathWorks, Inc., Natick, Massachusetts, United States) was used to postprocess and analyze the row and column signals obtained from our customized graphical user interface. As the computational time constraints of the live interface were no longer present, to enhance the precision of the center of tongue tip contact detection, we developed an edge-adjustment algorithm. This algorithm was employed when the center of the tongue tip contact was within 2.25 mm from the trackpad edge, we refer to these as partial contacts. For such contacts, the edge-adjustment algorithm utilized the size of the last complete contact to calculate the position of the center of the partial contact. Partial contacts with centers beyond the trackpad boundary were removed. Cubic smoothing splines were fitted to contact center positions. To account for contacts beyond the device boundary, breaks were included in the splines. The splines were filtered with a 5th order low-pass Butterworth filter with a cut-off frequency of 6 Hz. The splines were resampled at 180 Hz. Velocity and acceleration were derived from the position data. Two-dimensional probability distributions for all kinematic domains (position, velocity, acceleration) were created using a 20-by-20 bin histogram. The position histogram was bounded by the trackpad sensing area, while the velocity and acceleration histograms ranges were selected to accommodate ~ 80% of all subjects achieved range (velocity: -6 cm/s to 6 cm/s, acceleration: -90 cm/s^2^ to 90 cm/s^2^). A median filter was applied to enhance the color scale of the position histogram. To account for user-intended periods of inactivity, data points with a speed lower than 0.1 cm/s were eliminated. The cumulative representations of tongue movement across 10 min of free-exploration for two subjects representing each group are shown in Fig. 2A.

#### Time to characterization

To obtain a comprehensive understanding of the evolution of movement patterns for each subject the 10-minute free-exploration dataset was divided into 5-second epochs. Each epoch was randomly partitioned into a training (75%) and test (25%) dataset. Distributions of successive cumulative training datasets were compared to the entire cumulative test dataset using the Coefficient of Determination (CoD). For each subject, this randomization process was conducted 50 times, generating new training and test datasets for all kinematic domains. The point at which the CoD reached 0.90, the time to characterization, was calculated for each randomization step. Additionally, to evaluate chronological effect, the time to characterization was calculated using randomly ordered 5-second epochs. The time to characterization was compared between feedback conditions and between kinematic domains using a Two-Way Repeated Measures Analysis of Variance (2-way RM-ANOVA). The alpha level was set at 0.05 for all analyses. Post-hoc tests were conducted using the Bonferroni correction.

The time to characterization analysis revealed that 5 min of data would sufficiently capture subjects’ movement patterns across all kinematic domains (discussed in the Results section). Therefore, 5 min of data was used for all further analysis.

#### Intra-subject similarity

**Fig. 3:**
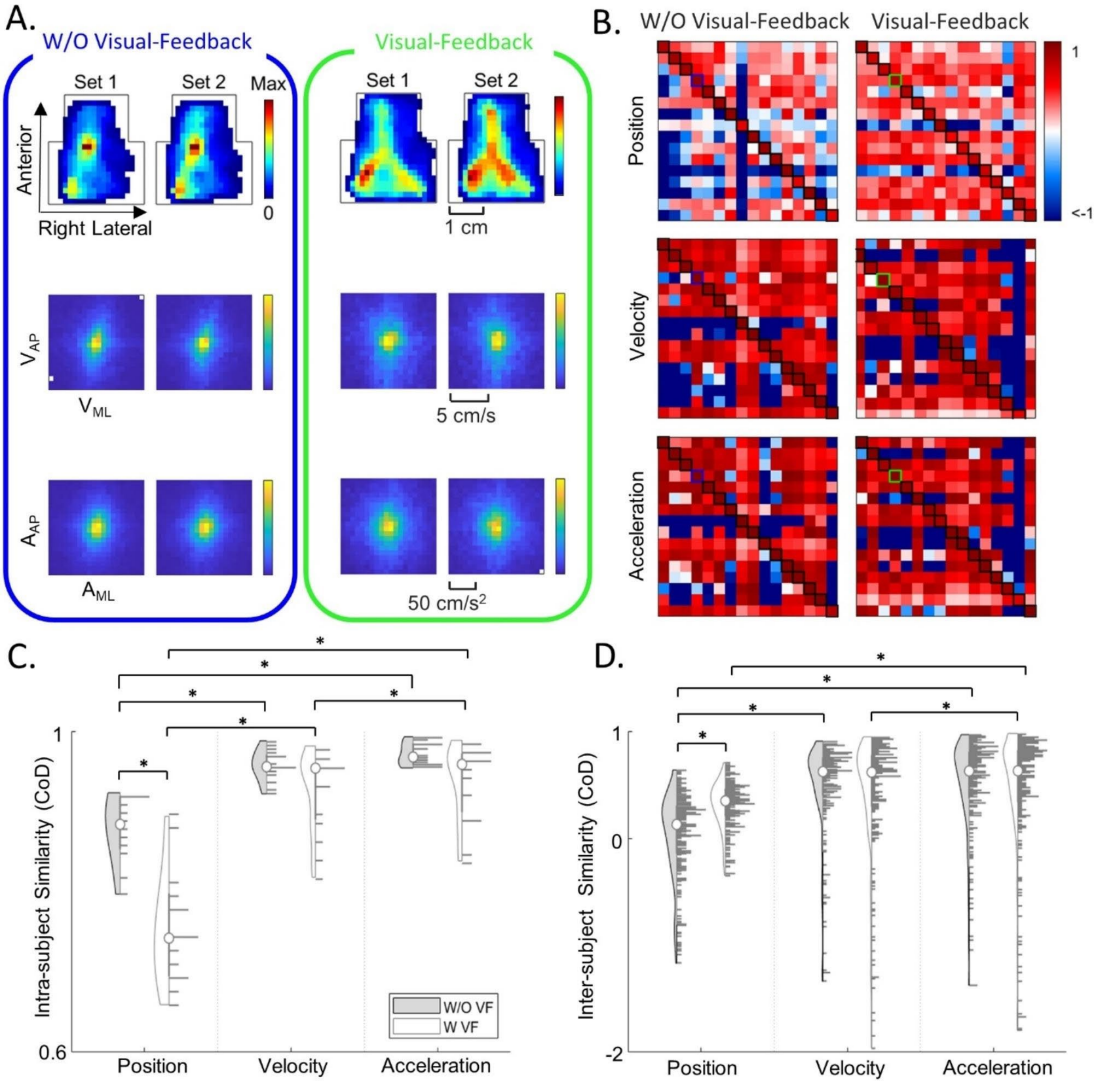
Intra- and Inter-Subject Similarity. **(A)** Probability distributions for a representative segment of randomly selected 5 min data (Set 1), along with the subsequent 5 min (Set 2). The blue and green boarders denote representative subjects from Fig. 2. **(B)** The average inter-subject confusion matrices, computed from 50 iterations, are presented across the three kinematic domains for both feedback groups. Rows denote a subject as a predictor, while columns represent a subject as the test data. The bolded diagonal elements indicate the intra-subject similarities between Set 1 and Set 2 (blue and green squares denote representative subjects in **(A)**). Violin plots illustrating the intra- subject **(C)** and inter-subject **(D)** similarity of data in **(B)**. The left side of each violin plot represents the data spread, while the right side shows a histogram of the data, and the white circle marks the median. Outliers, defined as points more than 1.5 interquartile ranges above the upper quartile or below the lower quartile, were excluded for visualization. Significant difference (*p* < 0.05) is indicated by *. These results suggest that intra-subject position distributions are less similar for the VF group, while inter-subject position distributions are more similar

To investigate the self-similarity of the free-exploratory task, a subjects’ distribution was compared to another 5-minute distribution of their own movement. Ten 30-second blocks of movement data were randomly selected to generate a 5-minute distribution (Set 1), and the remaining 5 min of data was assigned to Set 2 (Fig. 3A). To evaluate the similarity between the two distributions, the CoD was calculated between Set 1 and Set 2, using one as a predictor and the other as test, and vice versa. This randomization process was repeated 50 times, generating a total of 100 CoD values for each subject. The CoD values were then averaged to determine a score for intra-subject similarity. Statistical differences between feedback conditions and kinematic domains were analyzed using a 2-way RM-ANOVA (α = 0.05, post-hoc Bonferroni correction).

#### Inter-subject similarity

To examine inter-subject similarity, the degree to which one subject’s distribution predicted other subjects, from the same feedback group, was assessed. Ten 30-second blocks of movement were randomly selected to generate a 5-minute distribution for each subject. The CoD was computed for all subject pairs in the same group, resulting in a 16 × 16 inter-subject confusion matrix. The randomization process was repeated 50 times, and the inter-subject matrices were averaged to generate a score for each subject pair. These scores failed to meet the normality assumptions; hence non-parametric tests were selected to assess statistical differences. The Mann-Whitney U test was used to assess differences in between the two independent feedback groups. Additionally, as the kinematic domains (position, velocity, acceleration) were related measures, Friedman’s test was adopted to assess differences among them.

#### Movement expression

To assess movement expression across all kinematic domains, we examined coverage area and bivariate kurtosis of the distributions. We further analyzed position distributions by comparing them to a uniform distribution. Coverage area, a measure of range of movement, was determined by calculating the number of bins with values that fell above the 5th percentile. Bivariate kurtosis was computed using the method described by Mardia [36]. To compare the position distributions to a uniform distribution, the CoD between the two distributions was calculated. All metrics were computed for 50 permutations of 5 min of data, each composed of ten randomly selected 30-second blocks. The values of the 50 randomization steps were averaged to obtain a subject score for each metric. For coverage area, statistical differences across kinematic domain were compared between the feedback groups using a Student’s t-test (α = 0.05). Statistical differences in bivariate kurtosis among feedback groups and kinematic domains were analyzed using a 2-way RM-ANOVA (α = 0.05, post-hoc Bonferroni correction). The differences in position uniformity amongst feedback groups were assessed using a Student’s t-test (α = 0.05). Additionally, we investigated the impact of partial contacts and analyzed all the metrics (coverage area, bivariate kurtosis, and uniformity of the distributions) within the sensor region where the positional accuracy was less than 1 mm, referred to as *full-contact region.*

#### Capability reference generation

For each subject, free-exploration blocks of movement data were randomly partitioned into equal halves, with 50% allocated to training and the remaining 50% to test datasets. Within each feedback group, the position distributions of the training datasets were averaged. These averages were then utilized to predict the 16 test datasets in the representing feedback group using the CoD. This process resulted in 16 CoD values for each feedback group that were averaged to drive a mean CoD value, referred to as a *prediction score*. The entire randomization process and the prediction score calculation was iterated 50 times. The resulting 50 prediction scores were statistically compared using a Student’s t-test (α = 0.05) amongst the feedback groups. Subsequently, the training distributions of the feedback group exhibiting a significantly higher prediction score were averaged to generate a capability reference.

## Results

### Time to characterization

As mentioned in the Methods section, we observed that 5 min of data were sufficient to characterize movement distributions across all kinematic domains. As expected, coefficient of determination (between the training and cumulative test datasets) gradually increased over time, suggesting that a subjects’ movement distributions approached their overall pattern as more data was included (Fig. 2B). A 2-way RM-ANOVA revealed that the feedback conditions did not play a role in the time to characterization. However, the choice of kinematic domain played a significant role in the amount of data required to reliably characterize the movement distributions, with velocity reaching time to characterization the fastest (2-way RM-ANOVA, F (2, 62) = 20.11, *p* < 0.001). Post-hoc comparisons using Bonferroni’s correction showed that, on average, velocity was 61% faster than position in the W/O VF group and only 34% faster in the VF group (*p* < 0.001). As expected, as chronological effect was eliminated by randomizing the order of the epochs, the time to characterization decreased for all kinematic domains across both feedback groups (Fig. 2B, inserts). Feedback condition had a main effect on the time to characterization (2-way RM-ANOVA, F (1, 31) = 7.26, *p* = 0.009). Post-hoc analysis showed that the W/O VF group reached the time to characterization 29% faster than the VF group for position (*p* = 0.013). Kinematic domains also had a main effect on the time to characterization (2-way RM-ANOVA, F (2,62) = 27.68, *p* < 0.001), with velocity being 66% faster than position in the W/O VF group and 55% faster in the VF group (*p* < 0.001). The results indicate that, on average, all kinematic domains in both feedback groups reached the time to characterization in 5 min. Consequently, 5 min of free-exploration was determined as necessary duration to construct movement probability distributions. For a visual representation of the distributions for all subjects across all kinematic domains, refer to Fig. S1 (additional file 1).

### Intra-subject similarity

Interestingly, subjects were less repeatable in their free-exploration distributions across all kinematic domains in the visual feedback group. A two-way RM-ANOVA revealed significant differences in intra-subject similarity scores between the feedback groups (F (1,31) = 9.09, *p* = 0.004). Post-hoc comparisons using Bonferroni’s correction revealed that the W/O VF group achieved 12% higher similarity score for position than the VF group (*p* = 0.029) (Fig. 3B and C). Furthermore, differences in intra-subject similarity scores were observed across kinematic domains, with acceleration achieving the highest intra-subject similarity (2-way RM-ANOVA, F (2, 62) = 35.11, *p* < 0.001). On average, in the W/O VF group, acceleration exhibited a 16% higher intra-subject similarity score than position (*p* < 0.001). While in the VF group, acceleration achieved a higher intra-subject similarity score than both position and velocity by 24% and 2% respectively (*p* < 0.001).

### Inter-subject similarity

Providing visual feedback led to an increased similarity in tongue movement patterns. Mann-Whitney U tests indicated that the visual feedback significantly affected inter-subject similarity in position (z = -7.71, *p* < 0.001), with the VF group having a mean rank that was 51% higher than the W/O VF group (Fig. 3B and D). Non-parametric Friedman tests of differences among repeated measures revealed that the choice of kinematic domain significantly affected inter-subject similarity in both the W/O VF group (χ ^2^(2) = 76.23, *p* < 0.001) and the VF group (χ ^2^(2) = 22.16, *p* < 0.001). In the W/O VF group, the mean rank of position was 28% lower than that of velocity (*p* < 0.001) and 33% lower than that of acceleration (*p* < 0.001). In the VF group, the mean rank of position was 19% lower than that of acceleration (*p* < 0.001) and the mean rank of velocity was 10% lower than that of acceleration (*p* = 0.032). Higher similarity in higher kinematic domains could be attributed to constraints in the movement range. These results suggest that with visual feedback, tongue movement patterns in position became more similar within the group, which could lead to the development of a more consistent capability reference.

Interestingly, we observed that subjects are more similar to themselves than they are to others. When comparing intra- to inter-subject similarity, we found that intra-subject values were higher in the W/O VF group (median ± interquartile range, pos: 0.87 ± 0.11, vel: 0.96 ± 0.03, acc: 0.96 ± 0.03) and the VF group (pos: 0.74 ± 0.10, vel: 0.94 ± 0.08, acc: 0.95 ± 0.08) than the inter-subject values in the W/O VF group (pos: 0.07 ± 0.63, vel: 0.54 ± 0.85, acc: 0.56 ± 0.86) and VF group (pos: 0.33 ± 0.33, vel: 0.51 ± 1.12, acc: 0.52 ± 1.07).

### Movement expression

**Fig. 4:**
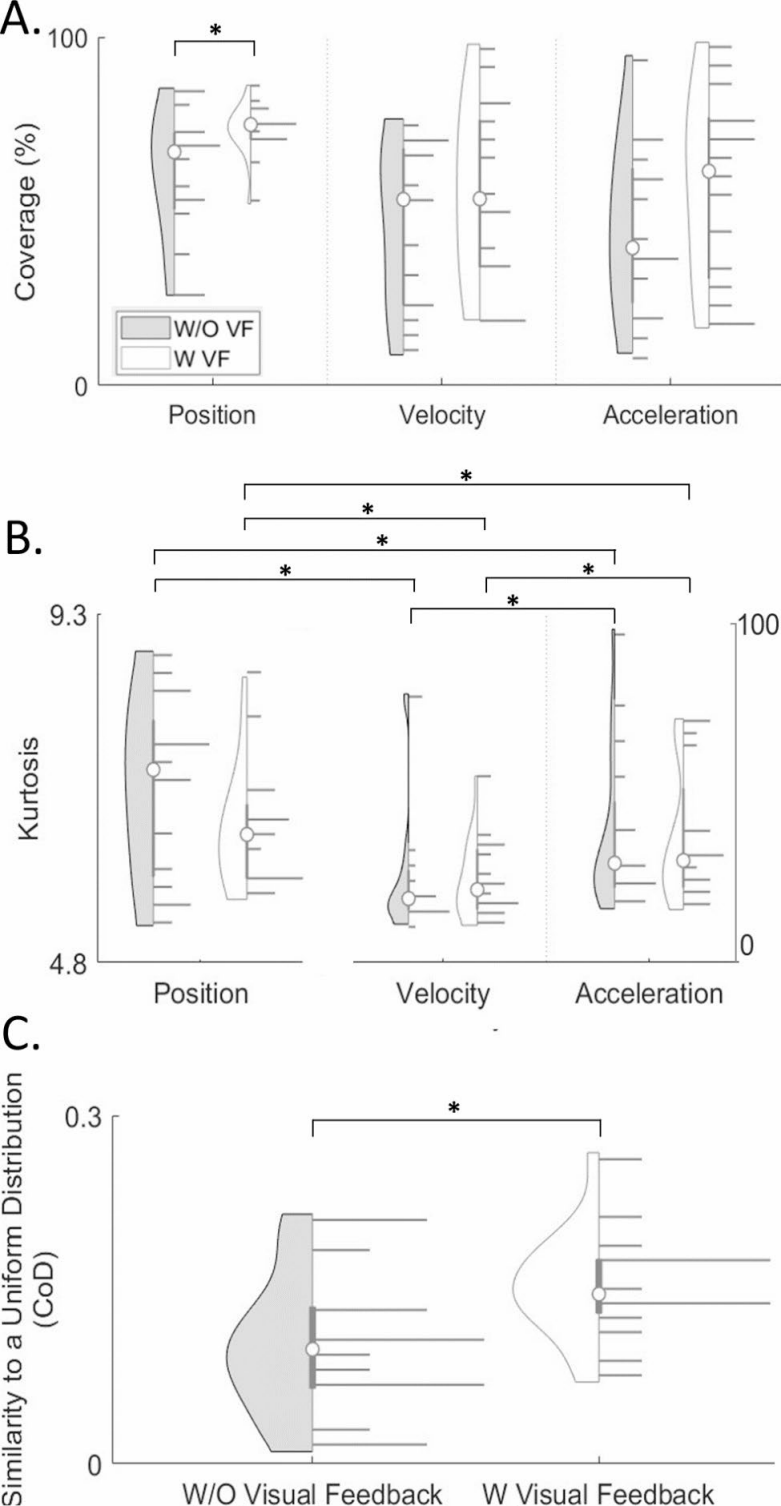
Movement Expression. Violin plots illustrate **(A)** coverage area and **(B)** bivariate kurtosis of position, velocity, and acceleration distributions for the W/O VF group (gray) and the VF group (white). **(C)** Violin plots display the comparison of the position distributions to a uniform distribution using CoD. The left side of each violin plot represents data spread, while the right side depicts a histogram of the data, with the median marked with a white circle. Significant differences (*p* < 0.05) are indicated by *. These results suggest that for the VF group, the coverage area in position increased, and the distribution became more uniform

By incorporating visual feedback, the explored positional area increased, and the distribution of movement became more uniform. The Student’s t-test revealed that the feedback conditions had a significant effect in the coverage area in position distribution with VF allowing for 22% greater coverage area (*p* = 0.04) (Fig. 4A). Interestingly, when limiting the analysis to the full-contact region, no significant difference in coverage area between feedback conditions was observed. This finding suggests that feedback encouraged the exploration of the trackpad edges.

While no significant difference in bivariate kurtosis was observed between feedback conditions, a significant difference between kinematic domains was found with position domain exhibiting the lowest bivariate kurtosis (2-way RM-ANOVA, F (2, 62) = 50.55, *p* < 0.001) (Fig. 4B). Post-hoc comparisons using Bonferroni’s correction revealed that in the W/O VF group, on average, position achieved a 73% and 81% lower bivariate kurtosis than velocity and acceleration, respectively (*p* < 0.001). Similarly in the VF group, position achieved a 73% and 82% lower bivariate kurtosis than velocity and acceleration, respectively (*p* < 0.001). This difference could be due to the limited range of movement in higher kinematic domains due to anatomical constraints. When restricting evaluation to the full-contact region, similar to the entire trackpad, position had significantly lower bivariate kurtosis compared to velocity and acceleration (2-way RM-ANOVA, F (2, 62) = 46.84, *p* < 0.001). Specifically, in both the W/O VF and VF groups, position had an average of 72% and 80–82% lower bivariate kurtosis than velocity and acceleration (*p* < 0.001).

When comparing position distributions to a uniform distribution a significant difference was detected between the two groups with the VF group being 51% more similar to uniform distribution than the W/O VF group (*p* = 0.04) (Fig. 4C). While in the full-contact region VF group is only 39% more similar to uniform distribution than the W/O VF group (*p* = 0.02). These results suggest that with visual feedback subjects expressed a broader range of movement that is more uniformly distributed.

### Capability reference generation

**Fig. 5:**
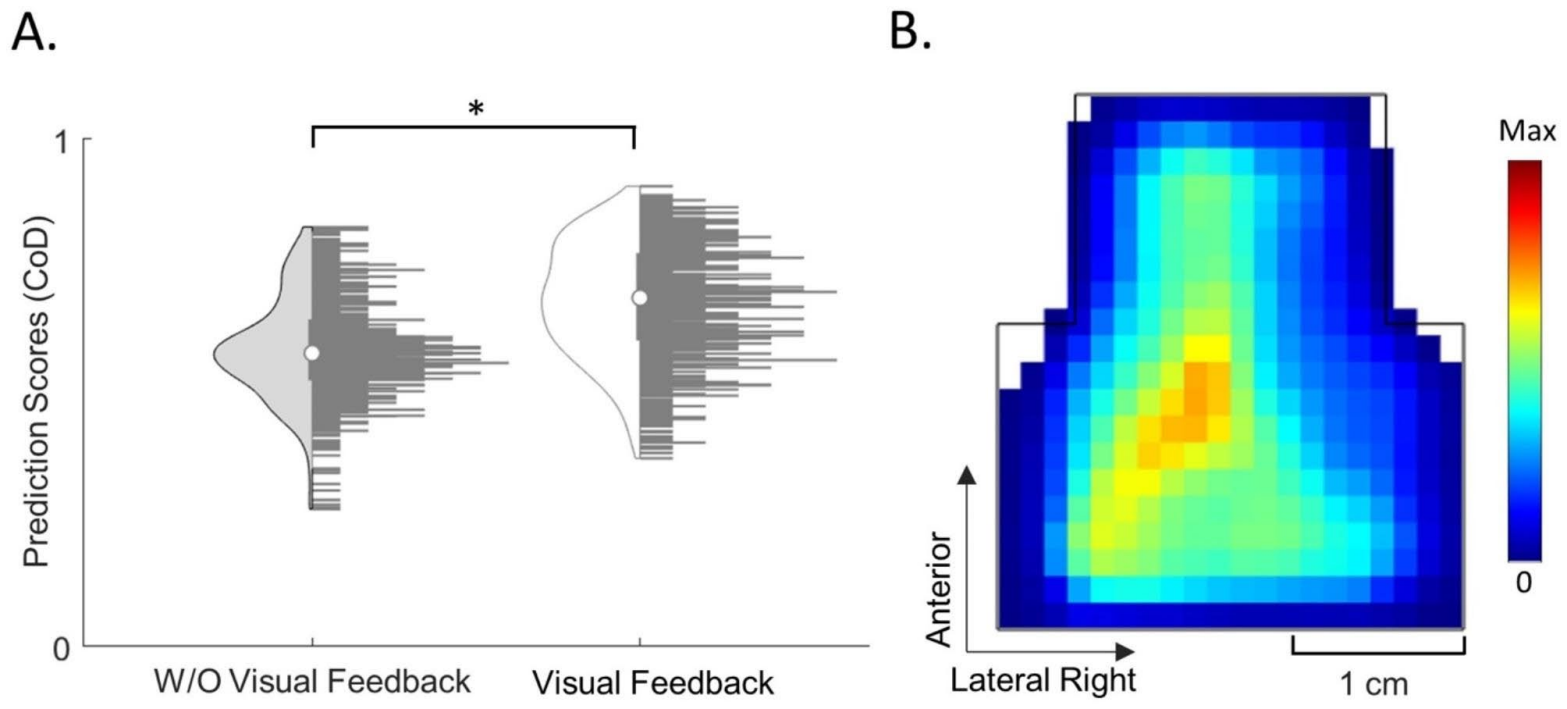
Capability Reference. **(A)** Violin plots depict the prediction scores for both the W/O VF and VF groups. The left side of each violin plot shows the data spread of the 50 iterations of prediction scores, while the right side displays a histogram, with the median marked by a white circle. Significant difference (*p* < 0.05) is indicated by *. **(B)** The capability reference was derived from the average of the VF group’s position probability distributions. Results indicate that the use of the VF group distributions significantly improved the prediction of new tongue movement distributions and enabled the development of a reference of capability

The incorporation of visual feedback in position training datasets resulted in higher predictability scores in the test datasets. These prediction scores were significantly higher than those in the W/O VF group (*p* < 0.001) (Fig. 5A). The mean prediction score for the VF group was 0.68 ± 0.11, a 42% increase over the W/O VF group’s mean score of 0.48 ± 0.32.

Given these results, with the VF group demonstrating higher inter-subject similarity, wider and more uniform distributions, and higher prediction scores, the position probability distributions were averaged to generate the capability reference (Fig. 5B).

## Discussion

We introduced a new methodology that consisted of the free-exploration task and the Tongue-Trackpad for studying tongue movement. We investigated the influence of visual feedback on tongue movement probability distributions. The major findings of this work indicate that with visual feedback, we observed: (1) higher inter-subject similarity, (2) a greater coverage area with a more uniform distribution, and (3) higher prediction ability of tongue distributions. These findings led to the development of a reference of unimpaired tongue capability.

In our evaluation of the amount of data required to characterize tongue distributions, we observed that when considering the temporal progression of tongue distributions, feedback conditions exhibited no impact on the time required for characterization. However, when mitigating the impact of chronological effects through the randomization of epoch orders, it became evident that the group receiving visual feedback required more time to achieve characterization in the position domain. A potential reason for this observation could be that providing visual feedback may have prompted individuals to employ adaptive strategies, resulting in a diverse array of movement patterns. This, in turn, may have required a greater volume of data to achieve characterization. However, in both chronological and randomized orders, velocity reached time to characterization faster than position under both feedback conditions. A possible explanation for this observation could be anatomical constraints, which may restrict the range of attainable tongue velocities. This, in turn, could influence the swifter time to characterization.

Our study revealed that subjects demonstrated intra-subject similarity in their distribution patterns, yet visual feedback had a diminishing effect on this similarity. It is important to note that in our analysis of similarity, we generated 5-minute distributions by randomly selecting ten 30-second exploration blocks. Hence, the random 30-second blocks held the potential to exhibit distinct movement behavior, as feedback may have encouraged subjects to diversify their movement to achieve the goal of covering the entire explorable area.

Also, we observed that intra-subject similarity was higher than inter-subject similarity for both feedback groups and across all kinematic domains. This finding suggests the presence of a unique, subject-specific distribution pattern that makes one more self-similar and less similar to others. Our data suggest that visual feedback increased inter-subject similarity in the position domain. This may indicate that subjects displayed distinctive, subject-specific patterns when feedback was absent, which may have diminished with feedback. Additionally, we observed that with feedback, subjects achieved a greater and more uniform coverage area, which also contributes to higher inter-subject similarity.

While the position distributions of subjects became more similar with visual feedback, no differences were observed in velocity and acceleration distributions. One potential reason for this observation could be the anatomical limitation on the achievable ranges of tongue velocity and acceleration. Another possible explanation could be that the user interface only provided subjects with a history of positional exploration and not a history of changes in velocity or acceleration. Future work could investigate whether providing feedback in the velocity or acceleration domains could influence the distributions of these domains.

As mentioned, an important finding was that the group receiving visual feedback expanded their explorable coverage area, and their distributions became closer to a uniform distribution. We further investigated this observation and examined the effect of partial contacts on the coverage area. Interestingly, we found no significant difference in coverage area between the feedback groups in the full-contact region. However, we observed that the feedback group exhibited a greater number of contacts at the edges, suggesting that visual feedback may have encouraged exploration of the edges. It is important to note that the developed user interface did not provide the subjects with feedback regarding the frequency of contact at various locations. In the future, exploring the potential impact of such feedback on the spread of the position distributions could be a valuable area of investigation.

We have also gained insight into our metrics and their utility. While evaluating feedback groups, bivariate kurtosis showed similar trends to the uniformity metric; however, a significant difference was not observed. Although previous researchers have found kurtosis to be a useful metric for evaluation of limb movement [37, 38].

It is interesting to note that experiments focused on the upper limb have illustrated that the velocity domain serves as a more effective discriminator, particularly between unimpaired individuals and those with impairments [2, 3, 39]. The absence of differentiation in velocity within our study may be attributed to the confined space of the oral cavity, which inherently restricts the range of attainable tongue velocities.

In addition, the capability reference we developed revealed a slight asymmetry in the distribution, with a bias toward the left side. However, this bias was not observed across all subjects (Fig. S1, additional file 1). Further studies will be necessary to explore the origin of this bias, which could have interesting implications for the neural motor pathways responsible for tongue movement. It should be noted that our non-parametric capability reference does not attempt to explain the underlying biomechanics or neurophysiology of tongue movement. Some may argue that a model of tongue structure and motor control would perform better at describing tongue movement. It is also important to note that this study was conducted in a parallel design, and employing a crossover experimental design could have allowed for investigation of design order effects.

Furthermore, the capability reference presented in this work may only be applicable to specific recording devices. We developed a device with a planar contact surface to avoid differences among different curvilinear palates. However, movement on a transverse plane does not reflect natural tongue movement as seen in vital tasks of speech, breathing, mastication, and swallowing. Future studies could explore self-directed tasks using curvilinear sensor arrays that allow additional ranges of tongue movement that might relate closer to the vital tasks. In this work, free-exploration was used to isolate the tongue and revealed some of its capabilities. Yet, the tongue is only a part of the variety of fine motor movements required for vital tasks as tongue movement in and of itself is not functional. We assert that by assessing tongue movement capabilities we provide a foundation for understanding only one critical component of these vital tasks. Other abilities of lip, jaw movement, strength, and coordination require attention.

## Conclusion

With the goal of developing a healthy tongue capability reference for tongue movement analysis, we introduced a new methodology involving the utilization of the free-exploration task and Tongue-Trackpad. Providing visual feedback during free-exploration resulted in higher inter-subject similarity, a broader coverage area, and greater uniformity in tongue movement probability distributions. These probability distributions were used to develop a reference of unimpaired tongue capability. This capability reference could lead to future developments in designing new interfaces, quantifying tongue ability, developing new diagnostic and rehabilitation techniques, and studying the underlying mechanisms of tongue control.

### Electronic supplementary material

Below is the link to the electronic supplementary material.

Supplementary Material 1. Additional File 1 contains Fig. S1 (.jpg), displaying the distributions of all subjects..

## Data Availability

The dataset generated and/or analyzed during the current study are available from the corresponding author upon reasonable request.

## Abbreviations

IOP: Iowa-Oral-Pressure-Instrument
EMA: Electromagnetic Articulography
EPG: Electropalatography (EPG)
PCB: Printed Circuit Board
BLE: Bluetooth Low Energy
CPU: Central Processing Unit
VF: Visual Feedback
W/O VF: Without Visual Feedback
HTML: HyperText Markup Language
CoD: Coefficient of Determination
RM-ANOVA: Repeated Measures Analysis of Variance

## References

[1] Sanders I, Mu L, Amirali A, Su H, Sobotka S. The human tongue slows down to speak: muscle fibers of the human tongue. Anat Rec. 2013;296(10). 10.1002/ar.22755 PMC3787083 23929762

[2] Huang FC, Patton JL. Movement distributions of Stroke survivors exhibit distinct patterns that evolve with training. J Neuroeng Rehabil. 2016;13(1). 10.1186/s12984-016-0132-y PMC4785660 26961682

[3] Huang FC, Patton JL. Augmented dynamics and motor exploration as training for Stroke. IEEE Trans Biomed Eng. 2013;60(3). 10.1109/TBME.2012.2192116 PMC4914037 22481803

[4] Wright ZA, Fisher ME, Huang FC, Patton JL. Data sample size needed for prediction of movement distributions. In: 2014 36th Annual International Conference of the IEEE Engineering in Medicine and Biology Society, EMBC 2014. 2014. 10.1109/EMBC.2014.6944941 PMC4936900 25571309

[5] Krakauer JW, Carmichael ST. Broken Movement. Broken Movement. 2019.

[6] Wright ZA, Patton JL, Huang FC. Energetics during robot-assisted training predicts recovery in stroke. In: Proceedings of the Annual International Conference of the IEEE Engineering in Medicine and Biology Society, EMBS. 2018. 10.1109/EMBC.2018.8512737 PMC8767422 30440917

[7] Park JS, Kim HJ, Oh DH. Effect of tongue strength training using the Iowa oral performance instrument in Stroke patients with dysphagia. J Phys Ther Sci. 2015;27(12). 10.1589/jpts.27.3631 PMC4713759 26834320

[8] Lin CH, Chung SY, Lin C, Te, Hwu YJ. Effect of tongue-to-palate resistance training on tongue strength in healthy adults. Auris Nasus Larynx. 2021;48(1). 10.1016/j.anl.2020.07.014 32727703

[9] Yang CC, Chung YM, Chi LY, Chen HH, Wang YT. Analysis of verbal diadochokinesis in normal speech using the diadochokinetic rate analysis program. J Dent Sci. 2011;6(4).

[10] Hewitt A, Hind J, Kays S, Nicosia M, Doyle J, Tompkins W et al. Standardized instrument for lingual pressure measurement. Dysphagia. 2008;23(1). 10.1007/s00455-007-9089-0 17602265

[11] Adams V, Mathisen B, Baines S, Lazarus C, Callister R. A systematic review and meta-analysis of measurements of tongue and hand strength and endurance using the Iowa Oral Performance Instrument (IOPI). Vol. 28, Dysphagia. 2013. 10.1007/s00455-013-9451-3 23468283

[12] E2 Scientific Corp. Swallowing Disorder Biofeedback at the Tip of the Tongue. 2023.

[13] Mori K, Manda Y, Kitagawa K, Nagatsuka H, Furutera H, Kodama N et al. Coordination of surface electromyography activity in the posterior tongue region during mastication of differently textured foods. J Oral Rehabil. 2021;48(4). 10.1111/joor.13135 33319400

[14] Perlman AL. Electromyography and the study of oropharyngeal swallowing. Dysphagia. 1993;8(4). 10.1007/BF01321778 8269730

[15] Davis JW, Lazarus C, Logemann J, Hurst PS. Effect of a maxillary glossectomy prosthesis on articulation and swallowing. J Prosthet Dent. 1987;57(6). 10.1016/0022-3913(87)90370-2 3473231

[16] Fukuoka T, Ono T, Hori K, Wada Y, Uchiyama Y, Kasama S et al. Tongue pressure measurement and videofluoroscopic study of swallowing in patients with Parkinson’s Disease. Dysphagia. 2019;34(1). 10.1007/s00455-018-9916-5 29948261

[17] Kwan BCH, Jugé L, Gandevia SC, Bilston LE. Sagittal Measurement of Tongue Movement during respiration: comparison between Ultrasonography and magnetic resonance imaging. Ultrasound Med Biol. 2019;45(4). 10.1016/j.ultrasmedbio.2018.12.003 30691918

[18] Stone M. A three-dimensional model of tongue movement based on ultrasound and x-ray microbeam data. J Acoust Soc Am. 1990;87(5). 10.1121/1.399188 2189921

[19] Zu Y, Narayanan SS, Kim YC, Nayak K, Bronson-Lowe C, Villegas B et al. Evaluation of swallow function after tongue cancer treatment using real-time magnetic resonance imaging: a pilot study. JAMA Otolaryngol Head Neck Surg. 2013;139(12). 10.1001/jamaoto.2013.5444 PMC5110428 24177574

[20] Rebernik T, Jacobi J, Jonkers R, Noiray A, Wieling M. A review of data collection practices using electromagnetic articulography. Vol. 12, Laboratory Phonology. 2021.

[21] Hardcastle W, Jones W, Knight C, Trudgeon A, Calder G. New developments in electropalatography: a state-of-the-art report. Clin Linguist Phon. 1989;3(1).

[22] Wood SE, Timmins C, Wishart J, Hardcastle WJ, Cleland J. Use of electropalatography in the treatment of speech disorders in children with Down syndrome: a randomized controlled trial. Int J Lang Commun Disord. 2019;54(2). 10.1111/1460-6984.12407 30039902

[23] Katz WF, Mehta S. Visual feedback of tongue movement for novel speech sound learning. Front Hum Neurosci. 2015;9(NOVEMBER). 10.3389/fnhum.2015.00612 PMC4652268 26635571

[24] Preston JL, Leaman M. Ultrasound visual feedback for acquired apraxia of speech: a case report. GPS Solutions. 2014;18(1).

[25] Hartelius L, Theodoros D, Murdoch B. Use of electropalatography in the treatment of disordered articulation following traumatic brain injury: a case study. J Med Speech Lang Pathol. 2005;13(3).

[26] Byun TMA, Hitchcock ER, Swartz MT. Retroflex versus bunched in treatment for rhotic misarticulation: evidence from ultrasound biofeedback intervention. J Speech Lang Hear Res. 2014;57(6). 10.1044/2014_JSLHR-S-14-0034 PMC4294189 25088034

[27] Blyth KM, McCabe P, Madill C, Ballard KJ. Ultrasound in dysphagia rehabilitation: a novel approach following partial glossectomy. Disabil Rehabil. 2017;39(21). 10.1080/09638288.2016.1219400 28029056

[28] Bressmann T, Harper S, Zhylich I, Kulkarni GV (2016). Perceptual, durational and tongue displacement measures following articulation therapy for rhotic sound errors. Clin Linguist Phon.

[29] Preston JL, McAllister T, Phillips E, Boyce S, Tiede M, Kim JS et al. Remediating residual rhotic errors with traditional and ultrasound-enhanced treatment: a single-case experimental study. Am J Speech Lang Pathol. 2019;28(3). 10.1044/2019_AJSLP-18-0261 PMC6802922 31170355

[30] Preston JL, Byun TM, Boyce SE, Hamilton S, Tiede M, Phillips E (2017). Ultrasound images of the tongue: a tutorial for assessment and remediation of speech sound errors. J Visualized Experiments.

[31] Hamed Mozaffari M, Guan S, Wen S, Wang N, Lee WS. Guided learning of pronunciation by visualizing tongue articulation in ultrasound image sequences. In: CIVEMSA 2018–2018 IEEE International Conference on Computational Intelligence and Virtual Environments for Measurement Systems and Applications, Proceedings. 2018.

[32] Barbier G, Merzouki R, Bal M, Baum SR, Shiller DM. Visual feedback of the tongue influences speech adaptation to a physical modification of the oral cavity. J Acoust Soc Am. 2021;150(2). 10.1121/10.0005520 34470311

[33] Marjanovic N, Piccinini G, Kerr K, Esmailbeigi H, TongueToSpeech. (TTS): Wearable wireless assistive device for augmented speech. In: Proceedings of the Annual International Conference of the IEEE Engineering in Medicine and Biology Society, EMBS. 2017. 10.1109/EMBC.2017.8037626 29060667

[34] Soresini G, Marjanovic N, Patton J, Esmailbeigi H. The Pattern of Tongue’s Motion: A Free-Exploration Study. In: Proceedings of the Annual International Conference of the IEEE Engineering in Medicine and Biology Society, EMBS. 2020. 10.1109/EMBC44109.2020.9176712 33018972

[35] Bratland V, Bondavalli D, Patton J, Esmailbeigi H. Movement of the Tongue During Target Reaching on a 2-Dimensional Surface. In: 2022 44th Annual International Conference of the IEEE Engineering in Medicine & Biology Society (EMBC). 2022. p. 4278–81. 10.1109/EMBC48229.2022.9871290 36085696

[36] Mardia Kv. Measures of multivariate skewness and kurtosis with applications. Biometrika. 1970;57(3).

[37] Vaisman L, Dipietro L, Krebs HI. A comparative analysis of speed profile models for wrist pointing movements. IEEE Trans Neural Syst Rehabil Eng. 2013;21(5). 10.1109/TNSRE.2012.2231943 PMC4689593 23232435

[38] Michmizos KP, Vaisman L, Igo Krebs H. A comparative analysis of speed profile models for ankle pointing movements: evidence that lower and upper extremity discrete movements are controlled by a single invariant strategy. Front Hum Neurosci. 2014;8(NOV). 10.3389/fnhum.2014.00962 PMC4245889 25505881

[39] Huang FC, Patton JL. Individual patterns of motor deficits evident in movement distribution analysis. In: IEEE International Conference on Rehabilitation Robotics. 2013. 10.1109/ICORR.2013.6650430 PMC4453863 24187248

