# Supplementary figures and images for: Statistical evaluation of tongue capability with visual feedback
Source: J Neuroeng Rehabil. 2024 Jan 2;21:2. doi: 10.1186/s12984-023-01293-7 (PMC10763040; doi:10.1186/s12984-023-01293-7)

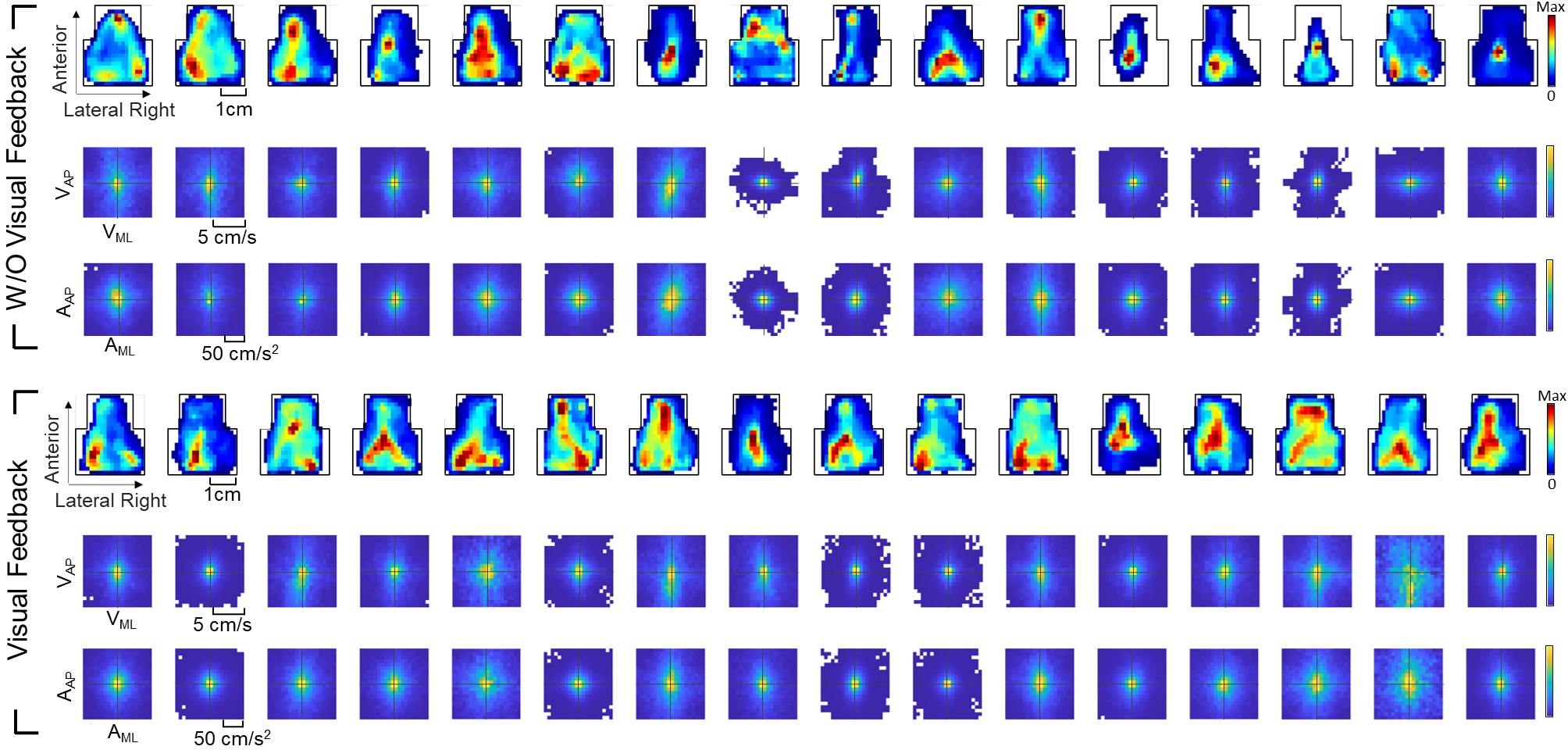

Supplement: Supplementary file 1 — Supplementary Material 1. Additional File 1 contains Fig. S1 (.jpg), displaying the distributions of all subjects.. [file 12984_2023_1293_MOESM1_ESM.jpg]
